# Metabolic regulation of stress erythropoiesis, outstanding questions, and possible paradigms

**DOI:** 10.3389/fphys.2022.1063294

**Published:** 2023-01-05

**Authors:** Baiye Ruan, Robert F. Paulson

**Affiliations:** ^1^ Pathobiology Graduate Program, Penn State University, University Park, PA, United States; ^2^ Center for Molecular Immunology and Infectious Disease, Department of Veterinary and Biomedical Sciences, Penn State University, University Park, PA, United States

**Keywords:** stress erythropoiesis, glycolysis, anabolic metabolism, epigenetic regulation, tissue regeneration

## Abstract

Steady state erythropoiesis produces new erythrocytes at a constant rate to replace the senescent cells that are removed by macrophages in the liver and spleen. However, infection and tissue damage disrupt the production of erythrocytes by steady state erythropoiesis. During these times, stress erythropoiesis is induced to compensate for the loss of erythroid output. The strategy of stress erythropoiesis is different than steady state erythropoiesis. Stress erythropoiesis generates a wave of new erythrocytes to maintain homeostasis until steady state conditions are resumed. Stress erythropoiesis relies on the rapid proliferation of immature progenitor cells that do not differentiate until the increase in serum Erythropoietin (Epo) promotes the transition to committed progenitors that enables their synchronous differentiation. Emerging evidence has revealed a central role for cell metabolism in regulating the proliferation and differentiation of stress erythroid progenitors. During the initial expansion stage, the immature progenitors are supported by extensive metabolic changes which are designed to direct the use of glucose and glutamine to increase the biosynthesis of macromolecules necessary for cell growth and division. At the same time, these metabolic changes act to suppress the expression of genes involved in erythroid differentiation. In the subsequent transition stage, changes in niche signals alter progenitor metabolism which in turn removes the inhibition of erythroid differentiation generating a bolus of new erythrocytes to alleviate anemia. This review summarizes what is known about the metabolic regulation of stress erythropoiesis and discusses potential mechanisms for metabolic regulation of proliferation and differentiation.

## Introduction

Steady state erythropoiesis maintains erythroid homeostasis, which requires precise control over the production and turnover of erythrocytes. The bone marrow has an enormous capacity for erythropoiesis and produces 2.5 × 10 ^ 6 erythrocytes per second in humans (Palis, 2014; Seu et al., 2017). This production is offset by a similar rate of turnover of erythrocytes in the spleen and liver such that the concentration of erythrocytes maintained in circulation optimizes oxygen delivery and minimizes problems due to increased blood viscosity (Klei et al., 2017). Situations that compromise oxygen delivery require a response that will increase tissue oxygenation. Blood loss and hypoxia can be compensated by increasing Epo levels which increases differentiation of committed erythroid progenitors (Tusi et al., 2018). In addition, sustained increases in serum Epo levels can skew hematopoiesis shunting progenitor cells down the CMP-MEP erythroid progenitor pathway at the expense of myelopoiesis (Grover et al., 2014; Singh et al., 2018). This phenomenon is enhanced steady state erythropoiesis. In contrast, inflammation caused by tissue damage or infection affects erythropoiesis at many levels. Pro-inflammatory cytokines inhibit steady state erythropoiesis (Rusten and Jacobsen, 1995; Zamai et al., 2000; Xiao et al., 2002; Tsopra et al., 2009). These factors also skew hematopoiesis towards myelopoiesis which increases the production of myeloid effector cells at the expense of steady state erythropoiesis (Oduro et al., 2012; Pietras et al., 2016; Pietras, 2017; Caiado et al., 2021). Pro-inflammatory cytokines like IL-6 increase the levels of hepcidin, which leads to sequestration of iron, making it unavailable for hemoglobin synthesis (Jurado, 1997; Cassat and Skaar, 2013; Soares and Weiss, 2015). Furthermore, inflammatory signals increase erythrocyte turnover, which further exacerbates the anemia (Libregts et al., 2011; Akilesh et al., 2019). To compensate for this loss in production, inflammation induces stress erythropoiesis (Paulson et al., 2020a). Unlike steady state erythropoiesis, the pro-inflammatory cytokines initiate stress erythropoiesis and promote the proliferation of immature progenitor cells (Bennett et al., 2019). The expansion of this progenitor population leads to increased production of erythroferrone (Erfe), which inhibits hepcidin expression so that once these progenitors start to differentiate, iron will be available for hemoglobin biosynthesis (Kautz et al., 2014a; Kautz et al., 2014b; Arezes et al., 2018). Furthermore, increased erythroid turnover by macrophages leads to heme dependent signaling, which increases production by macrophages of key factors like GDF15 and BMP4 that are required for stress erythropoiesis (Bennett et al., 2019). These observations support the idea that stress erythropoiesis is part of a coordinated inflammatory response, which allows bone marrow hematopoiesis to concentrate on the production of immune effector cells while erythroid homeostasis is maintained by extra-medullary stress erythropoiesis (Paulson et al., 2020a). In mice, this response is primarily in the spleen and liver. Stress erythropoiesis is highly conserved in humans (Xiang et al., 2015; Paulson et al., 2020b; Chen et al., 2020). Although the location of stress erythropoiesis in humans has not be definitively identified, there are many reports of extra-medullary stress erythropoiesis in anemia patients (for review see (Paulson et al., 2020b)).

Because of the stress response nature of stress erythropoiesis, the strategy for erythroid production is different from steady state erythropoiesis. As described above, steady state erythropoiesis constantly produces new erythrocytes. Stress erythropoiesis generates a bolus of new erythrocytes that maintain homeostasis until the source of the inflammation can be resolved (Perry et al., 2009; Paulson et al., 2020b). This response is more similar to stem cell-based tissue regeneration systems like those observed in muscle, lung and the intestinal epithelium (Asfaha, 2015; Tidball, 2017; Shen et al., 2022). These systems rely on tissue specific stem cells that are distinct from the stem cells that maintain tissue homeostasis. A common theme in tissue regeneration is that these stem cells respond to inflammatory signals which leads to proliferation of a transient amplifying population of progenitors that differentiates into mature cells that repair the damaged tissue. One of the better characterized regeneration systems is muscle regeneration, where satellite cells resident in the muscle are activated by inflammatory signals expressed by macrophages and infiltrating monocytes (Burzyn et al., 2013; Tidball, 2017; Scaramozza et al., 2019; Theret et al., 2019; Theret et al., 2022). These cells proliferate and differentiate to form new myotubes that repair the damaged muscle. The signals that drive the differentiation of myogenic precursors coincide with a switch from an inflammatory environment to a pro-resolving response. This example underscores the key role for inflammatory signals in initiating tissue regeneration, but also shows that resolving inflammatory signals plays a role in the transition to differentiation. Stress erythropoiesis shows a similar developmental trajectory. The initiation of stress erythropoiesis relies on inflammatory signals. Macrophage expression of TNFα, Interferon *γ* (Ifnγ) and IL-1β is transiently increased in the spleen following treatment with LPS and zymosan, which induces inflammatory anemia. *In vitro*, TNFα increases the proliferation of immature stress erythroid progenitors (SEPs) suggesting that transient TNFα signaling in SEPs drives their proliferation (Bennett et al., 2019). The mechanism by which TNFα promotes proliferation is not understood. At this stage, SEPs maintain stem cell characteristics, are capable of self-renewal, but do not differentiate (Harandi et al., 2010; Xiang et al., 2015). Analysis of SEPs showed that this transient amplifying population is heterogenous, made up of three distinct populations, with CD34^+^CD133+Kit + Sca1+ cells being the most immature, CD34^neg^CD133+Kit + Sca1+ being the intermediate, and CD34^neg^CD133^neg^Kit + Sca1+ being the most mature (Xiang et al., 2015). The transition from a proliferating SEP to a SEP committed to erythroid differentiation relies on erythropoietin (Epo) signaling in splenic macrophages, which alters the signals made by the niche. Epo signaling leads to a loss of pro-proliferative signals like canonical Wnts and an increase in signals that promote differentiation like Prostaglandin E2 (PGE_2_) (Chen et al., 2020). This change in signals is part of a change in the niche from a pro-inflammatory niche dominated by M1 macrophage type signals to a pro-resolving niche dominated by M2 macrophage like signals (Bennett et al., 2019). After this transition, SEPs lose their ability to self-renew and commit to differentiation, characterized by continued proliferation and subsequent entry into the terminal differentiation pathway (Harandi et al., 2010; Xiang et al., 2015). Although many of the signals that regulate stress erythropoiesis have been identified, the role of cellular metabolism and how these signals affect metabolism is largely unknown. In this review, we discuss what is known about metabolic regulation of stress erythropoiesis. We will identify open questions and discuss examples from other systems that illustrate potential paradigms for how changes in metabolism can regulate proliferation and differentiation of SEPs.

## Metabolic control of progenitor proliferation

For efficient stress erythropoiesis, immature progenitor populations must be expanded prior to the commitment to differentiation. This process requires the translation of proliferative signals, like Wnt2b and 8a and GDF15, into changes in metabolism that support proliferation and prevent changes in chromatin structure associated with the activation of the erythroid gene program.

Our previous work identified signaling pathways that are required for the expansion of immature SEP populations. The questions that remain are how these signals regulate metabolism and how changes in metabolism result in the proliferating, self-renewing, uncommitted SEP populations we observe *in vivo*. To address these questions, we must first acknowledge that metabolism regulates all cellular processes. It is often discussed as though the different metabolic processes are independent pathways, however, that is not how we should think about metabolism. As articulated by Murphy and O’Neill, a more correct way to think about metabolism is that metabolic pathways are interconnected such that changes in one metabolic pathway affect the flux of metabolites through other pathways (Murphy and O'Neill, 2020). In stress erythropoiesis, the initial stage requires the expansion of immature SEP populations. Signals that drive the proliferation of SEPs establish a metabolism that favors anabolic processes required to generate sufficient amino acids, lipids, nucleotides and other macromolecules to generate biomass for cell division. In cancer cells, this type of metabolism is referred to as aerobic glycolysis or the “Warburg effect”, which is dominated by glycolysis and is present in rapidly proliferating cell populations (Warburg, 1956; Lunt and Vander Heiden, 2011). Although glycolysis is higher than oxidative phosphorylation at these times, this type of metabolism can really be thought of as a highly efficient anabolic metabolism where glycolytic metabolites can be shunted into anabolic pathways like the Pentose phosphate pathway (PPP) and the serine glycine (Ser/Gly) pathway, which generate nucleotides, amino acids, metabolites used in 1-carbon metabolism and regenerate NADPH levels (Wise et al., 2008; Lunt and Vander Heiden, 2011; Le et al., 2012). A role for these pathways in stress erythropoiesis was shown by Oburoglu et al. who demonstrated that commitment of human CD34^+^ cells to the erythroid lineage required glutamine and the PPP. Inhibition of glutamine metabolism with the glutamine analog 6-diazo-5-oxo-L-norleucine (DON) blocked erythroid commitment (Oburoglu et al., 2014; Oburoglu et al., 2016). This effect could be rescued with exogenous nucleotides further demonstrating the need for nucleotide biogenesis in erythropoiesis. Although this work used *in vitro* human CD34^+^ cell cultures, the same group showed that *in vivo* stress erythropoiesis induced by phenylhydrazine treatment in mice was blocked by DON, while myelopoiesis increased in the spleen (Oburoglu et al., 2014; Oburoglu et al., 2016). In cancer cells, highly efficient usage of the PPP and Ser/Gly pathways relies on the establishment of metabolons, like the purinosome, which is a complex of at least 10 enzymes that uses metabolic channeling to drive *de novo* purine biosynthesis (An et al., 2008; Zhao et al., 2015; Pedley et al., 2022). It remains to be demonstrated whether signals that promote the proliferation of SEPs also drive the formation of the purinosome and other metabolons to increase metabolic efficiency. The ability of the purinosome to increase purine biosynthesis has the potential to profoundly impact overall cell metabolism and cell signaling as increased levels of guanine and adenine activate the mTorC1 pathway (Ben-Sahra et al., 2016; Emmanuel et al., 2017; Hoxhaj et al., 2017). mTorC1 is a central regulator of metabolism. Blocking mTorC1 activity compromises stress erythropoiesis and limits SEP proliferation (Knight et al., 2014). Furthermore, through the downstream activation of ATF4, mTorC1 can feed back to increase purine biosynthesis by increasing the expression of Mthfd2, an enzyme involved in the mitochondrial tetrahydrofolate cycle (Ben-Sahra et al., 2016). Like mTorC1, mutations in ATF4 severely compromise stress erythropoiesis (Masuoka and Townes, 2002). In addition to purine biosynthesis, mTorC1 also increases pyrimidine biosynthesis by phosphorylating CAD, the initial enzyme in the *de novo* pyrimidine biosynthesis pathway (Ben-Sahra et al., 2013; Robitaille et al., 2013). This modification also leads to increased rRNA and ribosomal biogenesis, which are needed to increase protein biomass during cell division (Claiborne et al., 2022). Although data support the role of mTorC1/ATF4 dependent control of metabolism in proliferating SEPs, further work is needed to understand how pro-inflammatory signals establish this anabolic metabolism and how other key stress erythropoiesis signals like canonical Wnts, GDF15 and BMP4 impact these metabolic pathways.

Proliferating SEPs are able to self-renew and maintain an immature phenotype characterized by the expression of stem cell genes and low-level expression of erythroid genes (Xiang et al., 2015). How does metabolic regulation maintain this immature cell state? In macrophages, inflammatory signals alter the TCA cycle. This alteration is referred to as a “broken TCA cycle” (Lampropoulou et al., 2016; O'Neill, 2015; Ryan and O'Neill, 2020). Metabolites like citrate and succinate are exported from the mitochondria and act in other pathways. Glutamine is converted to alpha-ketoglutarate (αKG), which can generate both citrate and succinate through oxidative and reductive pathways (Mullen et al., 2014). When citrate and succinate are exported from the mitochondria, they contribute both to the anabolic metabolism and affect gene expression (Mills and O'Neill, 2014; Ryan et al., 2019; Tannahill et al., 2013; Williams and O'Neill, 2018). Exported citrate is converted by ATP Citrate lyase (Acly) to oxaloacetate and acetyl-CoA. In cancer cells, the latter is used by histone acetyltransferases to maintain expression of glycolytic enzymes (Wellen et al., 2009). Acetyl-CoA is also used in lipogenesis, which supports cell division. Succinate on the other hand acts as an inhibitor of αKG dependent dioxygenases, which includes the family of proline hydroxylases (PHDs) that regulate the stability of hypoxia inducible transcription factors (Hifs) (Tannahill et al., 2013). The inhibition of PHDs increases hypoxia dependent glycolysis. Other enzymes that are inhibited by succinate include jumonji-domain histone deacetylases and the TET proteins that demethylate DNA. These data suggest that increased succinate plays a role in inhibiting differentiation of SEPs by preventing changes in DNA and histone methylation. Further work is needed to establish that metabolic control of epigenetics marks inhibits erythroid differentiation, while at the same time increasing the expression of genes that promote proliferation.

**FIGURE 1:**
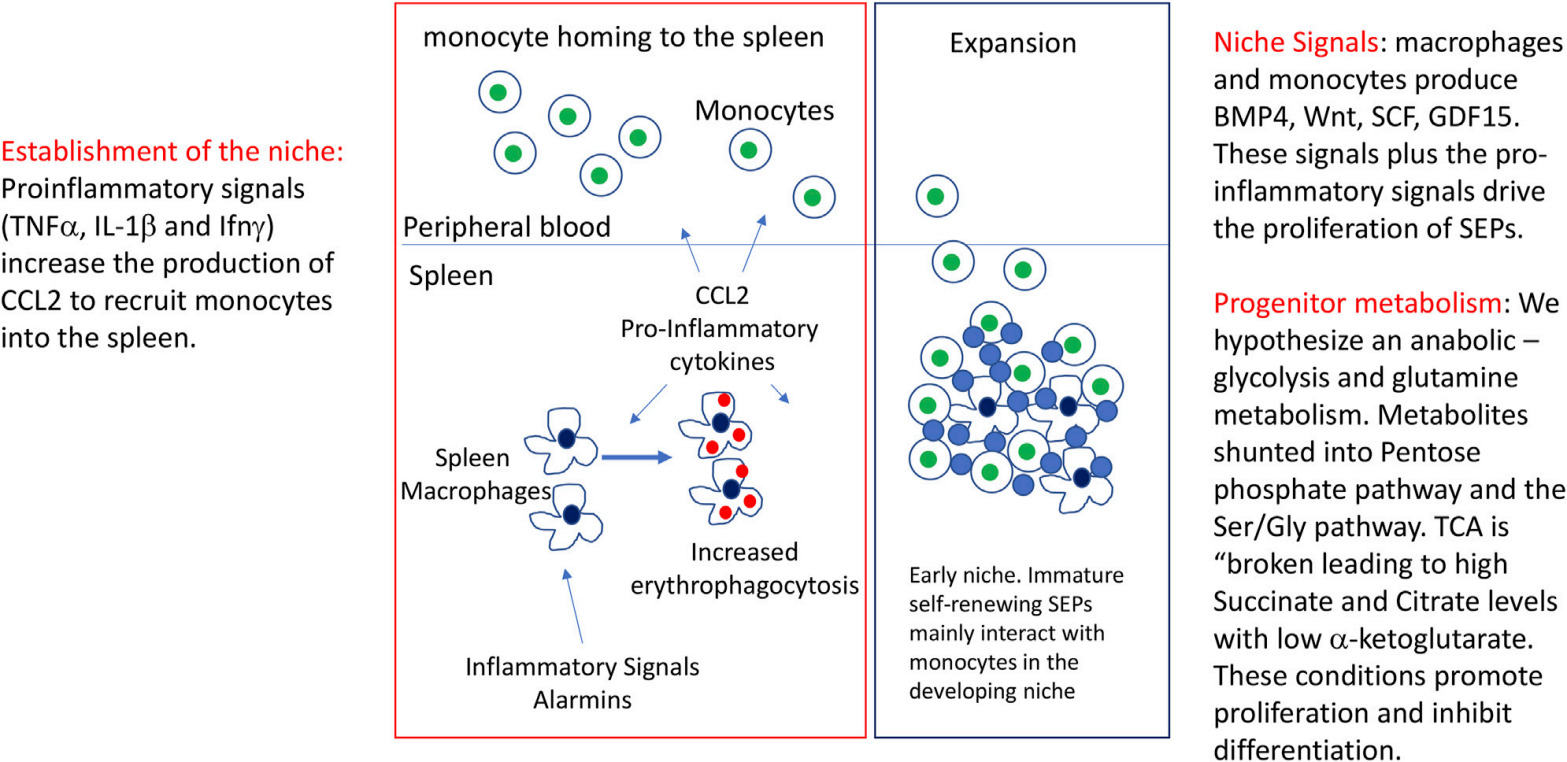
Initiation of stress erythropoiesis and the expansion of immature SEPs. Figure depicts the inflammatory signals that initiate stress erythropoiesis, the formation of the stress erythropoiesis niche and the expansion of a transient amplifying population of immature stress progenitors. Included in the figure are observed signals and potential changes that would be predicted to occur as discussed in the review.

GDF15 and the Hippo-Yap signaling pathways play an essential role in stress erythropoiesis (Hao et al., 2019a; Hao et al., 2019b)**.** Mutation of these pathways significantly impairs proliferation of SEPs. We observed that both pathways regulate the expression of genes involved in glycolysis and the TCA cycle (Hao et al., 2019b). GDF15 signaling increases the expression of PDK1 and PDK3, two enzymes that limit the flow of pyruvate into the TCA cycle, which is consistent with an altered TCA cycle in SEPs. GDF15 also increases the expression of Hif1α and Glut1, which increases glycolysis. Similarly, the expression of Gls1 is increased with GDF15 treatment. Gls1 is an enzyme that catalyzes the breakdown of glutamine, which channels glutamine into anabolic pathways. Like GDF15, our analysis of Yap1, a transcriptional co-activator whose activity is regulated by the Hippo pathway and Wnt signaling, showed that it maintains the proliferative anabolic metabolism in immature SEPs(Azzolin et al., 2014; Enzo et al., 2015; Koo and Guan, 2018; Hao et al., 2019a; Ibar and Irvine, 2020). Mutation of Yap1 severely impairs the expansion of immature SEP populations. Yap1 regulates the expression of Psat1, an enzyme in the Ser/Gly pathway which generates Serine from the glycolytic metabolite 3-phosphoglycerate that is used in one carbon metabolism and the production of S-adenosyl-methionine. Serine is also needed for the production of folate, which feeds into purine and pyrimidine biosynthesis. Yap1 also regulates the expression of Got1, a transaminase that generates aspartate from αKG. Inhibition of Got1 blocks erythroid commitment to the erythroid lineage (Hao et al., 2019a). In addition to our data, work in several experimental systems has shown thatYap1 regulates the expression of glycolytic enzymes and other enzymes involved in glutamine metabolism (Enzo et al., 2015; Cox et al., 2016; Cox et al., 2018; Koo and Guan, 2018; Ibar and Irvine, 2020). These data suggest that the mechanisms that regulate the proliferation of SEPs rely on a complex integration of signaling pathways which shape the anabolic metabolism needed to support the expansion of these progenitor populations. Further work is needed to understand how this integration of cell signaling and metabolism is accomplished. (Figure 1).

### Metabolic regulation of differentiation

**FIGURE 2:**
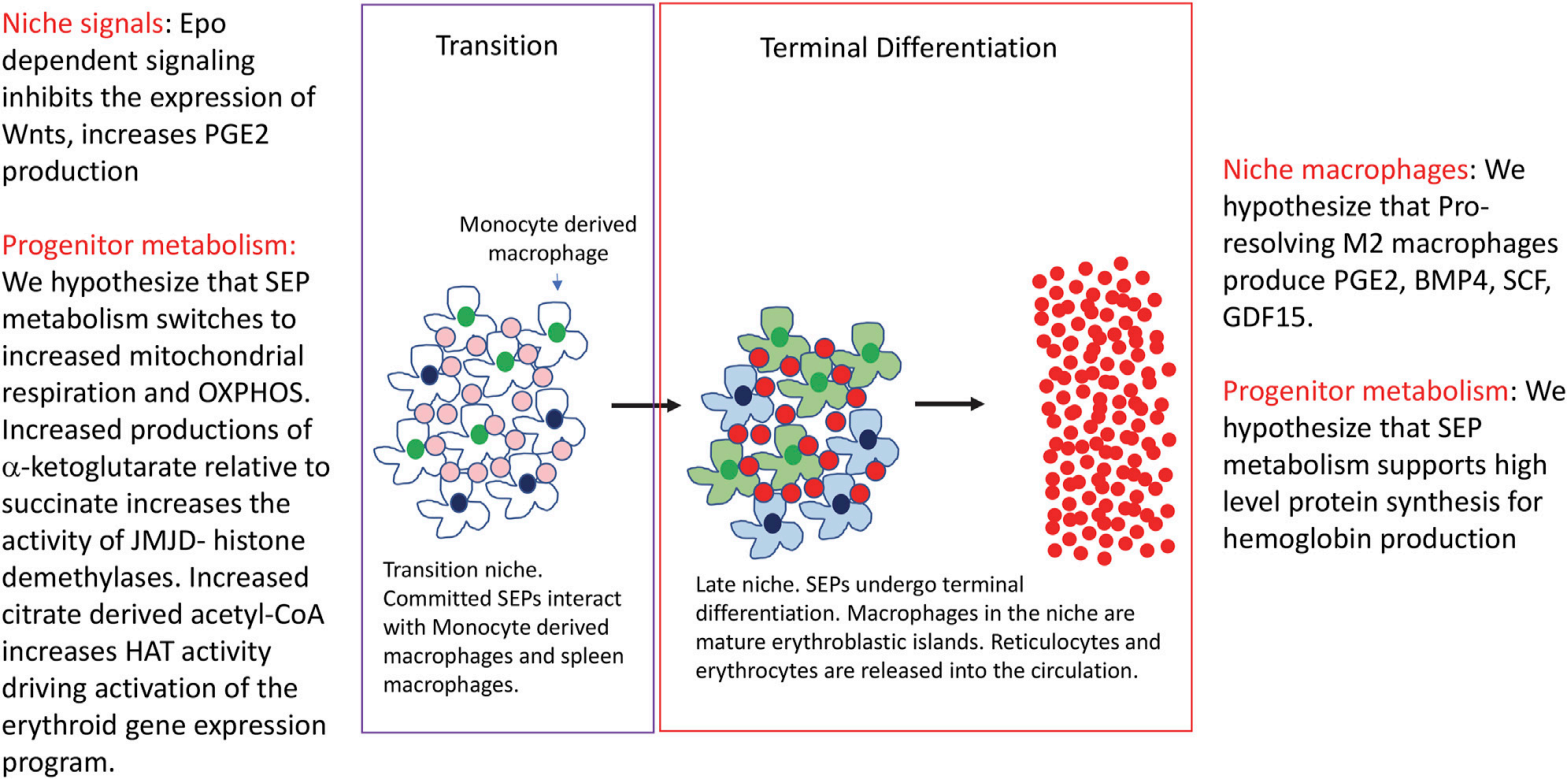
The transition to differentiation and the terminal differentiation of stress erythroid progenitors. The figure depicts the transition to differentiation and terminal differentiation. Changes in signals and metabolism in the niche and progenitors are indicated.

The transition from uncommitted proliferating SEPs to SEPs committed to differentiation requires changes in the signals made by the niche. Epo dependent signaling leads to a loss of Wnt expression and an increase in PGE_2_ production, however, GDF15, BMP4 and Yap1 dependent signaling pathways remain in SEPs, but now these pathways promote differentiation (Chen et al., 2020). In addition, the niche also switches from one dominated by pro-inflammatory signals to one dominated by pro-resolving anti-inflammatory signals (Liao et al., 2018). This change in signals and microenvironment will have a profound impact on the differentiation of SEPs. Although we know little about the metabolic changes that drive differentiation, data from others have identified some paradigms that may also play a role in stress erythropoiesis. (Figure 2). Analysis of hematopoietic stem cells (HSCs) showed that quiescent HSCs prefer glycolysis, but as they become mobilized and differentiate, oxidative phosphorylation (OXPHOS) increases (Simsek et al., 2010; Suda et al., 2011; Harris et al., 2013; Takubo et al., 2013; Ito and Suda, 2014; Maryanovich et al., 2015). Again, this switch in metabolism does not mean that glycolysis is off and OXPHOS is on, but rather the relative amounts of metabolites that flux through these pathways change. The increase of OXPHOS and the TCA cycle leads to changes in metabolites that affect other signaling pathways. As mentioned above, high levels of succinate generated when the TCA cycle is broken can inhibit jumonji-domain histone demethylases and TET DNA demethylases, but when mitochondrial respiration is increased and the levels of αKG available for enzymes increases. During muscle regeneration, the H3K27 demethylases JmjD3 and UTX (KDM6A), which require αKG, increase their activity at different times during the recovery from muscle injury (Liu and Rando, 2016; Gabellini, 2022). JmjD3 activity is needed to increase the expression of Has2, an enzyme that initiates the production of hyaluronic acid. The expansion of the extracellular matrix leads to an exit from quiescence allowing for the proliferation of muscle stem cells (Nakka et al., 2022). UTX has no role at this stage, however, later UTX is required for the commitment of myocytes to differentiation. This step is characterized by the UTX dependent removal of H3K27me3 at the myogenin locus and at other loci associated with muscle development. Mutation of JmjD3 or UTX leads to increased levels of H3K27me, but they show distinct defects in muscle regeneration (Faralli et al., 2016). These two examples demonstrate how tight regulation of histone H3K27 demethylase activity can control the proliferation and differentiation of muscle stem cells during muscle regenration. Changes in niche signaling can also induce metabolic reprogramming and induce histone demethylase activity. Germinal center B cell differentiation is induced by IL-4 signaling, which increases mitochondrial respiration and αKG production in activated B cells. By increasing this metabolite, UTX activity increases and removes H3K27me3 marks at the Bcl6 enhancer and promoter. In addition to changing metabolism, IL-4 signaling activates Stat6 which recruits UTX to the enhancer and promoter in Bcl6. These data show that IL-4 signaling establishes a metabolism that increases UTX activity, but also targets UTX to key genes required for germinal center B cell development (Haniuda et al., 2020). These examples show how changes in metabolism lead to mobilization of chromatin remodeling enzymes that drive the changes in gene expression programs necessary for the differentiation of progenitor cells. The specificity of these epigenetic changes comes from the integration of cell signaling with metabolic reprogramming. Future work on SEP differentiation will be needed to determine how pro-resolving signals lead to changes in metabolism that de-repress the erythroid gene expression program and promote differentiation.

The increase in αKG coupled with a relative decrease in succinate and fumarate will also lead to the activation of the Ten-eleven translocation (TET) enzymes. Like the histone demethylases these enzymes require iron, oxygen and αKG (Laukka et al., 2016; Koivunen and Laukka, 2018). They catalyze the oxidation of 5-methylcytosine (5-mC) to 5-hydroxymethylcytosine (5hmC), which can be subsequently converted to un-methylated cytosine (Cimmino et al., 2011; Wu and Zhang, 2011). Changes in DNA methylation have been previously shown to regulate globin gene expression (Ley et al., 1982; Ginder et al., 1998; Siegfried et al., 1999). In addition, analysis of human, mouse and zebrafish showed that Tet2 plays a role in lineage commitment. Analysis of human CD34^+^ bone marrow cell cultures, showed that there is an increase in 5hmC during the erythroid commitment stage of the culture, which was associated with increased binding of erythroid transcription factors and increased chromatin accessibility (Madzo et al., 2014). Cultures of cord blood cells showed that inhibiting Tet2 leads to increased myeloid differentiation, while decreasing erythroid differentiation (Pronier et al., 2011). ShRNA knockdown Tet2 in CD34^+^ cell cultures led to increased proliferation of immature erythroid progenitors and impaired their differentiation (Yan et al., 2017). These observations are of interest as mutations in TET2 are often observed in myelodysplastic syndromes, which exhibit impaired erythroid differentiation and is associated with clonal hematopoiesis (Fujishima et al., 2021; Florez et al., 2022; Gurnari et al., 2022). These data are similar to what is observed when Tet2 is knocked down in zebrafish embryos (Ge et al., 2014). These data suggest a role for TET2 dependent changes in DNA methylation in the commitment of SEPs to differentiation. Further work will be needed to determine how changes in DNA methylation affect stress erythropoiesis.

### Final thoughts

This small set of examples illustrate some of the areas where metabolism could regulate stress erythropoiesis. The challenge for future research will be to integrate transcriptomic and metabolomics data with cell signaling. These analyses will address how metabolism impacts gene expression at each of the developmental stages of stress erythropoiesis, how changes in metabolism drive the transition from proliferating SEPs to differentiating SEPs and how signals from the niche impact and change metabolism so that sufficient erythrocytes are produced to maintain erythroid homeostasis.
